# MicroShunt versus Trabeculectomy for Surgical Management of Glaucoma: A Retrospective Analysis

**DOI:** 10.3390/jcm11185481

**Published:** 2022-09-18

**Authors:** Michael X. Fu, Eduardo M. Normando, Sheila M. H. Luk, Mira Deshmukh, Faisal Ahmed, Laura Crawley, Sally Ameen, Niten Vig, Maria Francesca Cordeiro, Philip A. Bloom

**Affiliations:** 1 Department of Surgery and Cancer, Imperial College London, London SW7 2AZ, UK; 2 Nuffield Department of Medicine, University of Oxford, Oxford OX1 3SY, UK; 3 Imperial College Ophthalmology Research Group, Western Eye Hospital, London NW1 5QH, UK; 4 The Hillingdon Hospitals NHS Foundation Trust, Uxbridge UB8 3NN, UK

**Keywords:** glaucoma, surgical glaucoma treatment, filtration surgery, minimally penetrating glaucoma surgery

## Abstract

This case-control study aims to compare the efficacy, safety, and postoperative burden of MicroShunt versus trabeculectomy. The first consecutive cohort of MicroShunt procedures (n = 101) was matched to recent historical trabeculectomy procedures (n = 101) at two London hospital trusts. Primary endpoints included changes in intraocular pressure (IOP) and glaucoma medications. Secondary outcome measures included changes in retinal nerve fibre layer (RNFL) thickness, rates of complications, further theatre interventions, and the number of postoperative visits. From the baseline to Month-18, the median [interquartile range] IOP decreased from 22 [17–29] mmHg (on 4 [3–4] medications) to 15 [10–17] mmHg (on 0 [0–2] medications) and from 20 [16–28] mmHg (on 4 [3–4] medications) to 11 [10–13] mmHg (on 0 [0–0] medications) in the MicroShunt and trabeculectomy groups, respectively. IOP from Month-3 was significantly higher in the MicroShunt group (*p* = 0.006), with an increased number of medications from Month-12 (*p* = 0.024). There were greater RNFL thicknesses from Month-6 in the MicroShunt group (*p* = 0.005). The rates of complications were similar (*p* = 0.060) but with fewer interventions (*p* = 0.031) and postoperative visits (*p* = 0.001) in the MicroShunt group. Therefore, MicroShunt has inferior efficacy to trabeculectomy in lowering IOP and medications but provides a better safety profile and postoperative burden and may delay RNFL loss.

## 1. Introduction

Glaucoma is a leading cause of blindness worldwide [1]. Lowering the intraocular pressure (IOP) has long been held to be the only effective strategy to reduce glaucomatous progression [2]. In patients showing advancing visual field (VF) loss, inadequate IOP control, or experiencing side effects or non-adherence to medication, surgical intervention is often advocated to halt the disease progression [3]. Trabeculectomy with intraoperative mitomycin-C (MMC) has long been regarded as the gold standard surgical modality [4]. Despite well-documented IOP-lowering effectiveness and cost efficiency [5], trabeculectomy may be associated with lengthy postoperative care [4]. There is an increasingly perceived need for safer alternative surgical techniques, but also the recognition that any new procedure must be rigorously assessed for efficacy.

Several alternatives have been proposed, such as minimally invasive glaucoma surgeries (MIGSs). However, the IOP reductions achieved with MIGSs are modest and largely targeted at patients with mild-to-moderate glaucoma [6], often in combination with lens surgery. For more advanced glaucoma, minimally penetrating glaucoma surgeries (MPGSs) have demonstrated initial efficacy and safety [7], tending to reduce postoperative visits [8]. Compared to trabeculectomy, MPGSs used in the UK have increased significantly, particularly during the coronavirus pandemic [8]. The *ab interno* XEN^®^ Gel Stent (Allergan Inc., Irvine, CA, USA), although initially popular, is less commonly used due to early failure, necessitating numerous postoperative manoeuvres [9]. The *ab externo* PRESERFLO^TM^ MicroShunt (“MicroShunt,” Santen Pharmaceutical) is an alternative MPGS that has shown early promise [10].

Despite similarities between MicroShunt and trabeculectomy, such as subconjunctival dissection and MMC usage, MicroShunt implantation does not require scleral flap formation, sclerostomy, or iridectomy [7]. The posterior placement of the MPGS device may also influence the bleb position and morphology, which may affect issues such as the risk of bleb leaks, bleb-related infections, and bleb dysaesthesia; MicroShunts tend to drain more posteriorly, which may be associated with an improved safety profile. Controlling the aqueous outflow through flow resistance without relying on the precise suture-tensioning techniques used in trabeculectomy [11] may mitigate postoperative care and variations in surgical skill.

However, a paucity of literature compares MicroShunt to trabeculectomy, the gold standard [3,12,13]. A favourable safety profile was found with the MicroShunt, with conflicting evidence on its efficacy [3,12,13]. However, these studies had short follow-up periods of 6 months [3,13] and 12 months [12], with only one study investigating the VF mean deviation (VF/MD), and this was with 6-month data [3], which is insufficient to detect meaningful changes [14]. No previous studies have analysed the retinal nerve fibre layer (RNFL) thickness.

The purpose of this study was to compare MicroShunt to trabeculectomy as the primary glaucoma surgery in terms of efficacy, safety, and postoperative burden. To the best of our knowledge, this is the first study to compare a comprehensive set of outcome measures between both procedures in all glaucoma diagnoses, including RNFL thickness and number of postoperative visits, with an 18-month follow-up.

## 2. Materials and Methods

### 2.1. Study Design

This study adhered to the tenets of the Declaration of Helsinki and was given local regulatory approval. Consecutive MicroShunt procedures from August 2020 at the Imperial College Healthcare NHS Trust (ICH) and July 2021 at the Hillingdon Hospitals NHS Foundation Trust (THH) were identified from Medisoft electronic records and theatre records, corresponding to the initial procedure performed at each trust. Recent historical consecutive trabeculectomy procedures were also identified. Lists were then cross-checked against search-generated data obtained from Medisoft. If both eyes were eligible, the first operated eye was included [3,13]. Only patients undergoing primary incisional glaucoma surgery with a minimum 3-month follow-up until May 2022 were included. Patients were listed for both procedures based on surgeons’ discretion.

Using the case-control matching function in SPSS (IBM, Armonk, NY, USA, version 28.0.0), matching was performed using the variables’ age (with a ten-year match tolerance), sex, ethnic group, glaucoma diagnosis, and the first or second eye undergoing an IOP-lowering operation (where the first eye was operated before the observed timeframe). According to the available MicroShunt follow-up, identical follow-up durations were analysed for the corresponding trabeculectomy match. Patients who underwent post-procedural secondary IOP-lowering surgeries were analysed up until the point at which the intervening decision was made since the outcomes after secondary surgery no longer reflected the initial procedure. Manual data collection from clinic letters was cross-checked with search-generated data.

### 2.2. Surgery

All cases were performed by glaucoma consultants or fellows under direct supervision in both surgical centres. Similar standardised methods were used by all surgeons.

For trabeculectomy, access was obtained by the creation of a fornix-based conjunctival flap. Using a soaked sponge, 0.4 mg/mL MMC was applied for 3 min below Tenon’s capsule, followed by irrigation with 40 mL of sterile saline. After cautery, a rectangular partial-thickness scleral flap was created. A paracentesis was performed to allow anterior chamber (AC) refilling as required. The AC was entered with a sharp blade under the scleral flap, followed by sclerostomy and peripheral iridectomy. The scleral flap was closed with 2 releasable and 1 fixed 10-0 nylon sutures, and Tenon’s capsule and the conjunctiva were closed together using 10-0 nylon sutures.

For MicroShunt, following the creation of a fornix-based conjunctival flap, a soaked sponge with 0.4 mg/mL MMC was applied below Tenon’s capsule for 3 min, followed by irrigation with 40 mL of sterile saline. After cautery, the sclera was marked 3 mm from the limbus. A shallow scleral pocket was prepared with a 1 mm-width knife at the distally marked point. A needle was then used to create a transscleral tunnel from the apex of the scleral pocket into the AC to insert the MicroShunt, with the MicroShunt fin tucked tightly into the scleral pocket. Tenon’s and conjunctiva were then closed with 10-0 nylon sutures after verifying for continuous aqueous flow at the distal MicroShunt end.

The postoperative visits schedule for both procedures was guided by the department protocol, with modifications by the surgeons according to individuals’ needs. Visits closest to established time points were chosen to amalgamate heterogeneous data from both trusts [15]: Day-1, Week-1, Month-1, Month-3, Month-6, Month-12, and Month-18. Due to the paucity of data for other time points, the time points of 6, 12, and 18 months were chosen for VF measurements. Postoperative glaucoma medications (medications) were discontinued post-surgery immediately and reintroduced at surgeons’ discretion for inadequately controlled IOP.

### 2.3. Outcome Measures

Baseline demographics were recorded for the visit where patients were listed. Primary outcome measures included changes in IOP and medications. Secondary outcomes included: VF/MD, average RNFL thickness, success rates, complications and theatre interventions, duration of surgery, and the number of postoperative glaucoma visits.

In keeping with current standards [4,12,15], ‘complete success’ criteria were: [1] IOP ≤ 21 mmHg; [2] no further surgical reintervention for glaucoma; [3] no loss of light perception vision; [4] no chronic hypotony (IOP ≤ 5 mmHg at two consecutive follow-ups from Month-3); and [5] no usage of medications to maintain adequate IOP. Surgical interventions were those performed in an operating room setting, including needling. Qualified success followed the same ‘complete success’ criteria but allowed for the use of medications.

Since this study did not use washout IOPs and the baseline IOP was not significantly high for many patients with medications, defining success with an additional IOP percentage reduction was deemed unsuitable [3]. However, to enable comparability with other studies with differing definitions, ‘strict success’ was defined as the ‘complete success’ criterion but with a ≥20% IOP reduction compared to the baseline [3]. The three success criteria were also assessed using 18 mmHg and 14 mmHg as the upper limit of the criteria.

### 2.4. Statistical Methods

Data were entered into Excel (Microsoft Corp.,Washington, DC, USA, version 16.60) and analysed using GraphPad Prism (LLC, Manhattan, KS, USA, version 9.3.1). Histograms and Shapiro–Wilk tests confirmed non-normal distributions for every outcome except VF/MD. Continuous data were reported as median [interquartile range (IQR)] or mean ± standard deviation and categorical variables as proportions. Mann–Whitney U/unpaired *t*-tests (group comparisons) and Wilcoxon/paired *t*-tests (longitudinal samples) were performed for continuous variables. Fisher’s exact and Chi-squared tests compared categorical data. Probabilities of success were analysed using Kaplan–Meier survival curves. As matched patients had identical follow-up duration, loss to follow-up was not a censoring criterion, but failure at one visit was a censoring criterion. Two-sided *p* ≤ 0.050 was considered statistically significant.

## 3. Results

### 3.1. Baseline Characteristics

Included were 202 eyes from 202 patients, with 101 eyes in each group. Table 1 shows the baseline comparisons between the groups. The MicroShunt group had higher American Society of Anaesthesiology (ASA) grades than the trabeculectomy group. At months 6, 12, and 18, respectively, 142, 92, and 36 eyes were available for analysis.

### 3.2. Primary Outcomes

#### 3.2.1. IOP

**Figure 1:**
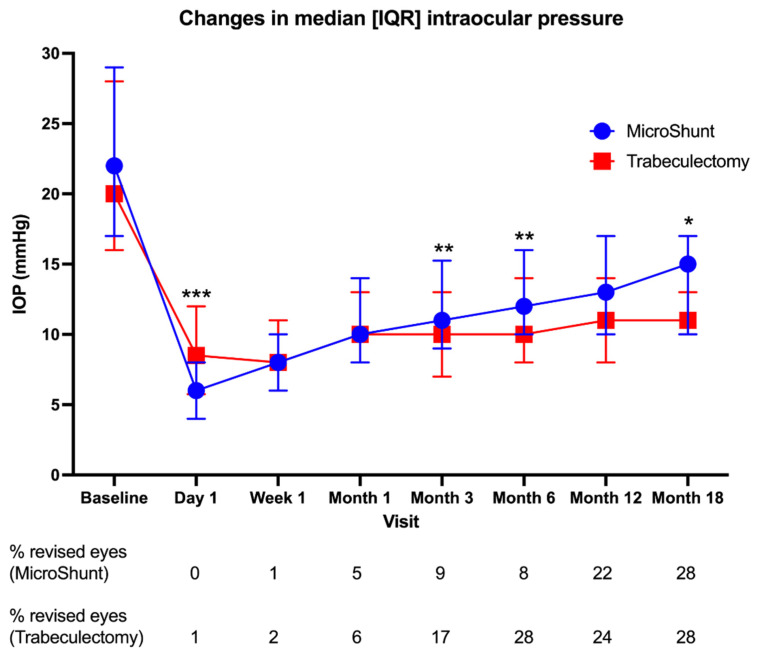
Changes in median IOP during follow-up after MicroShunt and trabeculectomy (* denotes *p* ≤ 0.050 between groups; ** denotes *p* ≤ 0.010 between groups, *** denotes *p* ≤ 0.001 between groups, assessed using Mann–Whitney U tests). Error bars represent interquartile ranges. The cumulative percentage of eyes undergoing bleb revision for each group as the proportion of the total number of available eyes for analysis is displayed under each time point.

IOP was significantly lower than the baseline at all time points (*p ≤* 0.001) for both groups. In the MicroShunt group, IOP decreased from 22 [17–29] mmHg at the baseline to 15 [10–17] mmHg at Month-18 (Figure 1). In the trabeculectomy group, IOP decreased from 20 [16–28] at the baseline to 11 [10–13] mmHg. On Day-1, the MicroShunt group had lower IOPs than the trabeculectomy group (6 [4–8] mmHg vs. 8 [5–12] mmHg, respectively, *p* < 0.001). At Month-3 (11 [9–15] mmHg vs. 10 [7–13] mmHg, *p* = 0.006) and Month-6 (12 [10–16] mmHg vs. 11 [8–14] mmHg, *p* = 0.048), the IOP in the MicroShunt group was higher, although the Month-12 IOP was still higher but not statistically significant (13 [10–16] mmHg vs. 11 [8–14] mmHg, *p* = 0.183). Month-18 IOP was also higher in the MicroShunt group but not statistically significant (*p* = 0.060). Figure 1 shows a trend toward a higher median IOP with time in the MicroShunt compared to the trabeculectomy group. When comparing the cumulative number of eyes undergoing bleb revision as a proportion of the total number of available eyes for analysis between the groups, this was only significantly more in the trabeculectomy at Month-6 (*p* = 0.004).

#### 3.2.2. Medications

**Figure 2:**
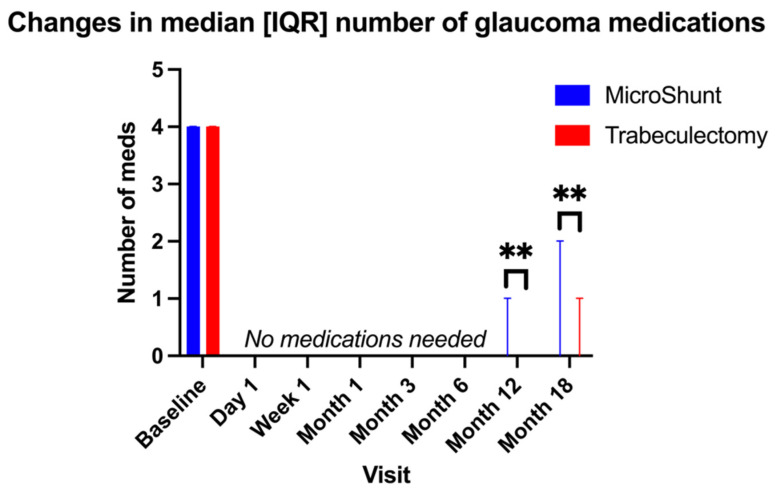
Changes in the median number of glaucoma medications during follow-up after MicroShunt and trabeculectomy (** denotes *p* ≤ 0.010 between groups, assessed using Mann–Whitney U tests). Error bars represent interquartile ranges.

Both procedures were associated with a significantly lower need for medications than at the baseline for all time points (*p* < 0.001). Medications were reduced from four [3–4] at the baseline to zero [0–0] in both groups immediately post-surgery (Figure 2), with no significant differences between the groups until Month-12, when the trabeculectomy group used fewer medications than the MicroShunt group (0 [0–0] vs. 0 [0–1], respectively, *p* = 0.024), continued at Month-18 (0 [0–0] vs. 0 [0–2], *p* = 0.019).

### 3.3. Secondary Outcomes

#### 3.3.1. VF/MD

The VF/MD was similar to the baseline at Month-6 and Month-12 in both groups but worsened at Month-18 compared to the baseline, with no differences between the groups at any time point (Table 2). A sub-analysis of pseudophakic and non-pseudophakic eyes showed similar results; however, the VF/MD was similar to the baseline at Month-18 in the non-pseudophakic MicroShunt eyes.

#### 3.3.2. RNFL Thickness

The average RNFL thicknesses transiently increased postoperatively in both groups (Table 2) and returned to baseline levels per Month-1. At Month-3, the average thicknesses were lower than the baseline in both groups. However, thicknesses in the MicroShunt group were comparable to the baseline from Month-6, whereas thicknesses continued to be lower than the baseline in the trabeculectomy group. Month-6 and Month-12 thicknesses were lower in the trabeculectomy group compared to the MicroShunt group.

#### 3.3.3. Success

**Figure 3:**
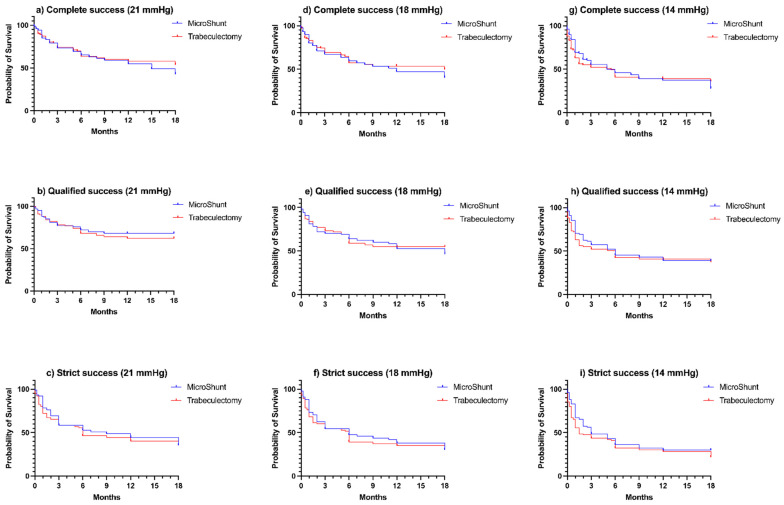
Kaplan–Meier survival curve of both groups during follow-up after MicroShunt and trabeculectomy: (**a**) complete success (IOP ≤ 21 mmHg; no theatre reoperation for glaucoma; no loss of light perception vision; no chronic hypotony (defined as IOP ≤ 5 mmHg on 2 consecutive follow-up visits from 3 months); and no use of postoperative adjunct medications to maintain adequate IOP), (**b**) qualified success (the aforementioned criteria, but with use of postoperative medications), (**c**) strict success (‘complete success’ criteria with at least 20% IOP reduction from baseline), (**d**) same as ‘(**a**)’ but with IOP ≤ 18 mmHg, (**e**) same as ‘(**b**)’ but with IOP ≤ 18 mmHg, (**f**) same as ‘(**c**)’ but with IOP ≤ 18 mmHg, (**g**) same as ‘(**a**)’ but with IOP ≤ 14 mmHg, (**h**) same as ‘(**b**)’ but with IOP ≤ 14 mmHg, and (**i**) same as ‘(**c**)’ but with IOP ≤ 14 mmHg. Log-rank (Mantel–Cox) test *p*-values were (**a**) *p* = 0.715, (**b**) *p* = 0.595, (**c**) *p* = 0.489, (**d**) *p* = 0.645, (**e**) *p* = 0.828, (**f**) *p* = 0.494, (**g**) *p* = 0.616, (**h**) *p* = 0.464, and (**i**) *p* = 0.464.

Using the 21 mmHg upper limit, the probabilities of complete success were 65.3%, 55.1%, and 42.8% for MicroShunt and 63.9%, 58.1%, and 53.9% for trabeculectomy at Month-6, Month-12, and Month-18, respectively. Figure 3 shows a divergence between the groups with time, with lower complete success in the MicroShunt group. The probabilities of qualified success were 72.0%, 68.2%, and 68.2% for MicroShunt and 68.1%, 62.1%, and 62.1% for trabeculectomy at Month-6, Month-12, and Month-18, respectively. The probabilities of strict success were 52.9%, 44.5%, and 35.6% for MicroShunt and 46.4%, 40.1%, and 35.6% for trabeculectomy at Month-6, Month-12, and Month-18, respectively. No significant differences were found between the groups for all the success criteria. With 18 mmHg and 14 mmHg upper limits, similar results were obtained.

#### 3.3.4. Complications and Interventions

Although more trabeculectomy than MicroShunt eyes had complications, this was not statistically significant between the groups (Table 3). The hypotony rates were similar, but there were more incidences of hypotony in the trabeculectomy group from Month-3 of the follow-up. Rates of chronic hypotony and maculopathy were higher in the trabeculectomy group. Ten trabeculectomy eyes required laser suture lysis.

Significantly more trabeculectomy eyes had theatre interventions. Bleb revision was the most common intervention in both groups, with more trabeculectomy eyes requiring this procedure. Two MicroShunt eyes required further IOP-lowering surgery.

#### 3.3.5. Operation Time and Number of Visits

The MicroShunt group had significantly shorter operation times (50 [44–62] vs. 71 [59–87] minutes, respectively, *p* < 0.001) and statistically significant fewer postoperative visits (8 [6–10] vs. 10 [7–13] visits, *p* = 0.001) than the trabeculectomy group.

## 4. Discussion

This study has found that trabeculectomy reduced the IOP and the need for medications more than MicroShunt. Changes in the VF and success rates were similar, but RNFL’s thickness loss slowed after MicroShunt. While complication rates were comparable, there were fewer theatre interventions and postoperative visits post-MicroShunt. These findings have a significant clinical impact, demonstrating that MicroShunt may not be the worthy successor to trabeculectomy in the treatment paradigm of moderate-to-advanced glaucoma.

### 4.1. IOP

Although IOP reaches low values in both groups, this study found that trabeculectomy had better IOP-lowering efficacy. The greater immediate IOP-lowering efficacy of the MicroShunt on Day-1, also reported in prior studies [16,17], may be attributable to the device’s predictable nature against trabeculectomy’s complex suture-tensioning techniques. There is clinical significance to the noticeable trend towards higher IOPs in the MicroShunt group over time. The non-significance between groups from Month-12 may be explained by the smaller sample sizes at each subsequent follow-up visit after Month-3, although this study’s sample size at Month-18 is comparable to previous retrospective studies with 6-month follow-up [3,13].

Baker et al.’s [12] study with 0.2 mg/mL MMC observed IOP reductions from 21.1 mmHg to 14.3 mmHg in the MicroShunt group at Month-12. We found a greater reduction at Month-12 from 22 [17–29] mmHg to 13 [10–16] mmHg. This suggests that 0.2 mg/mL MMC may be less efficacious for IOP-lowering than the 0.4 mg/mL in this study. Wagner et al.’s [13] study used 0.2 mg/mL MMC but found a similar IOP reduction as this study, suggesting that MMC concentration may have a minimal effect on efficacy. These discrepancies necessitate further investigations into the optimal MMC dose during glaucoma surgery. However, differences in the cohorts’ demographics, such as fewer ethnic heterogeneity [12] and medication use [12,13] than in this study, decrease scarring risk and subsequent IOP control [12] and thus may confound MMC comparisons. Nonetheless, the clinical significance of the greater IOPs from Month-3 and the trend toward greater IOP increases in the MicroShunt group found in this study constitutes a significant finding, suggesting that trabeculectomy should still be utilised as the preferred surgery when a lower target pressure is sought, such as those patients with normal-tension glaucoma. Although this study is the first to present Month-18 results comparing both procedures, longer-term investigations are required to investigate whether IOP continues to increase after Month-18 following MicroShunt when compared to trabeculectomy since trabeculectomy is known to lower IOP effectively over up to 20 years [18].

### 4.2. Medications

Although both groups significantly reduced medication use, greater medication-lowering efficacy was observed with trabeculectomy at Month-3, Month-12, and Month-18. The reductions are higher than in previous studies; for example, Baker et al. [12] reported reductions from 3.1 to 0.6 medications at Month-12 in their MicroShunt groups, compared to 4 [3–4] to 0 [0–1] medications in this study. The higher reductions found in this study may be due to higher baseline medications, enabling the medication-lowering efficacy of both procedures to be more pronounced. Our findings differ from previous comparative studies [3,12,13] that showed similar medication-lowering efficacy of MicroShunt and trabeculectomy. Whether this is due to conflicts of interest in previous studies on the MicroShunt [3,12] or small sample sizes [3,13], future prospective studies are necessitated to investigate whether the increase in medications following the MicroShunt procedure is a longer-term effect.

### 4.3. VF/MD

The VF/MD was not different between the groups at any time point, suggesting similar effects on the functional preservation effect by both surgeries. In the only previous study comparing VF between MicroShunt and trabeculectomy, Pillunat et al. [3] also reported no significant change in either group at Month-6. However, their small sample size of 26 eyes in each group limits the reliability of the conclusions. Furthermore, the inclusion of solely Caucasian patients invalidates the comparison with the present study, which included ≥45.5% non-Caucasians, who have a higher likelihood of subsequent failure after bleb-forming surgery than Caucasians [12]. Studies evaluating VF post-trabeculectomy have come to inconsistent conclusions [19,20], necessitating more studies comparing VF progressions after MicroShunt and trabeculectomy. Furthermore, the VF progression seen at Month-18 in both groups was likely due to natural glaucomatous progression despite IOP-lowering surgery or due to the decreases in the sample size with time. This may also explain the similarity of the VF/MD at Month-18 compared to the baseline in non-pseudophakic eyes in the MicroShunt group, suggesting that potential cataract progression may not have modulated VF/MD progression.

### 4.4. RNFL Thickness

The RNFL thickness after MicroShunt implantation has not been studied before. This study shows that MicroShunt may delay RNFL thickness loss compared to trabeculectomy. Similar to our findings, a previous study showed that RNFL thicknesses increased in the first postoperative week following trabeculectomy but decreased to baseline levels in subsequent visits [21]. Potential explanations include the reversal or rebound of the bowing of the laminar cribrosa by the elevated pre-intervention IOP, which manifests as a decreased cup area postoperatively [21]. It may also be due to the transient rebound to the normal shape and size of the retinal ganglion cell axons following dramatic IOP reductions post-surgery [22]. Additionally, Ch’ng et al. [23] attributed transient RNFL thickness increases post-trabeculectomy to postoperative inflammation. 

The thinner RNFL values in this study’s trabeculectomy cohort from Month-6 may be explained by the higher hypotony levels from the Month-3 follow-up observed with significantly more cases of chronic hypotony and maculopathy, where the formation of retinal folds may have decreased the RNFL thicknesses in the trabeculectomy cohort compared to the MicroShunt. Furthermore, it may have been difficult to measure RNFL thinning with a baseline median RNFL thickness of 57 μm and 55 μm in the MicroShunt and trabeculectomy groups, respectively, since there may be a ‘floor effect’ at approximately 50 μm with Spectralis OCT [24].

### 4.5. Success Rates

The trend toward lower ‘complete success’ in the MicroShunt group is clinically important, supporting that MicroShunt is clinically less efficacious than trabeculectomy. With similar definitions, Wagner et al. [13] also found no differences in ‘complete’ and ‘qualified’ successes. However, ‘strict success’ was significantly higher in their trabeculectomy group, and they observed higher success rates in both groups compared to the present study. This may be because of their small sample size and better baseline VF/MD, affecting comparability. Baker et al. [12] also observed lower ‘strict success’ in their MicroShunt than trabeculectomy groups (53.9 vs. 72.7%; *p* < 0.01) at Month-12. The prevention of medication reintroduction until a predetermined IOP threshold should have resulted in lower success rates than in the present study, where medications were reintroduced at the surgeons’ discretion. However, this study observed similar ‘strict success’ at Month-12 between the groups. This may be due to Baker et al.’s [12] baseline IOP inclusion threshold of ≥15 mmHg. Therefore, attaining a prespecified 20% IOP reduction may be easier compared to this study, which included all baseline IOP values. Their failure definition of inadequate IOP reduction at two consecutive visits compared to one in this study may also favour higher success. Additionally, their inclusion of solely primary open-angle glaucoma [12] may not reflect real-world results with differing glaucoma subtypes.

This study’s trabeculectomy ‘strict success’ rate at Month-18 (35.6%) was low compared to Kirwan et al.’s [25] 65% at Year-2. Many factors contribute to this difference: Patients had no previous MIGS or diodes, suggesting milder glaucoma, whilst a large proportion of our cohort had these procedures. Additionally, the inclusion of both eyes from each patient, where applicable, may have skewed their results in favour of higher success [26] and violated statistical assumptions [27]; the inclusion of more Caucasians (79% vs. 40.6% in this study) who have a lower risk of scarring after bleb-forming surgery than black patients [12]; and the inclusion of solely open-angle glaucoma.

### 4.6. Complications and Theatre Interventions

Although the lower complication rate in the MicroShunt group is statistically insignificant, this is still clinically important, and the lower theatre intervention rate for MicroShunt confirms the hypothesis of a better safety profile. Additionally, higher ASA grades in the MicroShunt group, which may have been intentional due to a better-hypothesised speed and safety, may have skewed safety profiles in favour of trabeculectomy.

The posterior MicroShunt bleb has a broad, diffuse, and low elevation with low vascularity, whereas the typical trabeculectomy bleb is anterior with a medium-high diffuse elevation and variable vascularity. Thus, the trabeculectomy bleb is more prone to over-draining and leakage (increasing the risk of infection and bleb dysaesthesia), resulting in more observed cases of chronic hypotony compared to the MicroShunt, which has a controlled lumen size that drains fluid more controllably. This theory makes it plausible that there were fewer postoperative theatre interventions in the MicroShunt group, as found by prior studies [3,12]. Indeed, due to the more problematic bleb, more cases of surgical bleb revision occurred in the trabeculectomy group. Differing definitions of what constituted a ‘theatre intervention’ and heterogeneity in the reporting hindered comparability with previous studies. Similar to Baker et al.’s [12] findings, laser suture lysis was necessary following trabeculectomy to allow for flow titration. The controlled flow titration of the MicroShunt eliminated the requirement for flow-restricting sutures and subsequent suture lysis.

### 4.7. Postoperative Visits and Operation Times

As the first study to compare postoperative visits, the findings confirm the hypothesis that MicroShunt results in less postoperative burden. Additionally, despite the potential learning curve with the new MicroShunt technique [12], a shorter MicroShunt operation time was found, contrary to Pillunat et al.’s [3] findings. The larger heterogeneous sample size supports the increased validity of the present study’s result. In the context of a significant backlog of glaucoma surgery after the coronavirus pandemic, the reduced operation time, visits, and reinterventions for the MicroShunt cohort mean that this device does constitute a favourable option for managing certain glaucoma patients. Furthermore, these factors may also mitigate the initial MicroShunt acquisition cost compared to the traditional trabeculectomy approach with minimal material costs and could be more cost-effective for lower-risk patients to manage glaucoma more safely. However, a recent cost analysis of Baker et al.’s [12] findings in American healthcare suggests that trabeculectomy is more cost-effective than MicroShunt [28], necessitating the validation of this finding in the UK NHS.

## 5. Conclusions

For the surgical treatment of refractory glaucoma in a clinically heterogeneous population, MicroShunt has inferior efficacy to trabeculectomy regarding IOP and medications, but MicroShunt has a superior safety profile and reduced postoperative burden. This contributes cautious findings to the paucity of evidence surrounding the MicroShunt’s potential to replace trabeculectomy as the gold standard operation in the glaucoma treatment paradigm, suggesting that trabeculectomy should still be reserved for higher-risk or more advanced patients. However, this study has also shown that the MicroShunt can reduce IOP safely with a potentially lesser burden on already stretched glaucoma services. Further longer-term studies are needed to corroborate this study’s findings and ascertain MicroShunt’s position in the surgical treatment of glaucoma.

## Figures and Tables

**Table 1:** Baseline demographics and characteristics. Data are expressed as proportions, means ± standard deviation, or medians [interquartile range] where appropriate. For the type of medications, ethnic groups, type of glaucoma, previous procedures, co-morbidities, and lens status, data represent the number of patients; ‘-’ denotes matched variables.

Patient Characteristics	MicroShunt	Trabeculectomy	*p*-Value
Age (years)	69 [57–78]	66 [57–76]	0.250 *
IOP (mmHg)	22 [17–29]	20 [16–28]	0.182 *
Number of medications	4 [3–4]	4 [3–4]	0.273 *
- Prostaglandins	94 (93.1%)	96 (95.0%)	0.767 #
- Beta-blockers	76 (75.2%)	85 (84.2%)	0.161 #
- Alpha-2-agonists	53 (52.5%)	63 (62.4%)	0.200 #
- Carbonic anhydrase inhibitors	91 (90.1%)	92 (91.1%)	>0.999 #
- Parasympathomimetics	0 (0.0%)	1 (1.0%)	>0.999 #
Ethnic group			-
- White	41 (40.6%)	41 (40.6%)	
- Black	17 (16.8%)	17 (16.8%)	
- Asian	14 (13.9%)	14 (13.9%)	
- Other	15 (14.9%)	15 (14.9%)	
- Not stated	14 (13.9%)	14 (13.9%)	
Identify as female	37 (36.6%)	37 (36.6%)	-
Identify as male	64 (63.4%)	64 (63.4%)	-
Best-corrected visual acuity (logMAR)	0.2 [0.0–0.5]	0.2 [0.0–0.5]	0.814 *
Visual field mean deviation (dB)	−13.35 ± 8.10	−14.38 ± 8.13	0.546 **
Average RNFL thickness (microns)	57 [47–71]	55 [47–68]	0.670 *
Type of glaucoma			-
- Primary open-angle glaucoma	74 (73.3%)	74 (73.3%)	
- Angle-closure glaucoma	12 (11.9%)	12 (11.9%)	
- Secondary open-angle glaucoma	14 (13.9%)	14 (13.9%)	
- Normal tension glaucoma	1 (1.0%)	1 (1.0%)	
Previous laser treatment	37 (36.6%)	29 (28.7%)	0.294 #
- Selective laser trabeculoplasty	13 (12.9%)	7 (6.9%)	0.238 #
- Cyclodiode laser	7 (6.9%)	8 (7.9%)	>0.999 #
- Micropulse diode laser	14 (13.9%)	10 (9.9%)	0.515 #
- Laser peripheral iridotomy	8 (7.9%)	6 (5.9%)	0.783 #
- Endoscopic Cyclophotocoagulation	1 (1.0%)	0 (0.0%)	>0.999 #
Previous MIGS	12 (11.9%)	10 (9.9%)	0.822 #
- iStent	8 (7.9%)	5 (5.0%)	0.568 #
- Cypass	4 (4.0%)	6 (5.0%)	0.748 #
First or second eye undergoing glaucoma surgery			-
- First eye	77 (76.2%)	77 (76.2%)	
- Second eye	24 (23.8%)	24 (23.8%)	
Co-morbidities			
- Diabetes	20 (19.8%)	22 (21.8%)	0.863 #
- Hypertension	48 (47.5%)	42 (41.6%)	0.479 #
- Anti-coagulant use	3 (3.0%)	2 (2.0%)	>0.999 #
ASA grade			0.030 +
- Grade 1	18 (17.8%)	34 (33.7%)	
- Grade 2	69 (68.3%)	53 (52.5%)	
- Grade 3	13 (12.9%)	13 (12.9%)	
Pseudophakic lens status	51 (50.5%)	41 (40.6%)	0.220 #

* Mann–Whitney U test; ** unpaired *t*-test; # Fisher’s exact test; ^+^ Chi-squared test.

**Table 2:** Changes in mean ± standard deviation VF/MD and median [IQR] RNFL thicknesses during follow-up after MicroShunt and trabeculectomy (* Mann–Whitney U test; ** unpaired *t*-test; ^#^ Wilcoxon test, ^##^ paired *t*-test).

	MicroShunt	*p*-Value (Comparison with Baseline)	Trabeculectomy	*p*-Value (Comparison with Baseline)	*p*-Value (Comparison between Groups)
Baseline					
VF MD (dB)	−13.35 ± 8.10	-	−14.38 ± 8.13	-	0.546 **
RNFL (μm)	57 [47–71]	-	55 [47–68]	-	0.670 *
Day-1					
RNFL (μm)	59 [46–78]	<0.001 #	59 [49–75]	0.002 #	0.795 *
Week-1					
RNFL (μm)	64 [52–75]	<0.001 #	56 [46–78]	0.011 #	0.151 *
Month-1					
RNFL (μm)	56 [49–71]	0.732 #	56 [48–68]	0.181 #	0.947 *
Month-3					
RNFL (μm)	56 [46–66]	0.012 #	54 [47–65]	<0.001 #	0.663 *
Month-6					
VF MD (dB)	−13.80 ± 7.53	0.668 ##	−14.47 ± 8.56	0.147 ##	0.735 **
RNFL (μm)	57 [48–66]	0.072 #	50 [44–55]	<0.001 #	0.005 *
Month-12					
VF MD (dB)	−14.52 ± 8.18	0.348 ##	−15.73 ± 7.39	0.192 ##	0.638 **
RNFL (μm)	55 [47–69]	0.114 #	50 [43–55]	0.003 #	0.046 *
Month-18					
VF MD (dB)	−15.97 ± 8.26	0.009 ##	−16.45 ± 8.08	0.026 ##	0.882 **
RNFL (μm)	52 [46–63]	0.101 #	51 [46–61]	0.016 #	0.674 *

**Table 3:** Numbers and proportions of patients with complications and interventions in both groups (* includes a patient with two occurrences, ** includes one patient with three occurrences, *** includes two patients with two occurrences, **** includes one patient with 5 occurrences).

Complication/Intervention	MicroShunt n (%)	Trabeculectomy n (%)	*p*-Value
Hypotony	46 (45.5%)	51 (50.5%)	0.573
- <3-months	44 (43.6%)	50 (49.5%)	0.890
- ≥3-months	4 (4.0%)	16 (15.8%)	0.008
Chronic hypotony	0 (0.0%)	10 (9.9%)	0.002
Choroidal effusion	13 (12.9%)	15 (14.9%)	0.839
Choroidal detachment	0 (0.0%)	2 (2.0%)	0.498
Hypotony maculopathy	1 (1.0%)	9 (8.9%)	0.019
Hyphaema	17 (16.8%)	11 (10.9%)	0.309
Malignant glaucoma	1 (1.0%)	0 (0.0%)	>0.999
Laser suture lysis	0 (0.0%)	10 (9.9%) *	0.002
Flat AC	4 (4.0%)	6 (5.9%)	0.748
AC reformation in theatre	0 (0.0%)	4 (4.0%) *	0.121
AC washout in clinic	1 (1.0%)	0 (0.0%)	>0.999
AC washout in theatre	2 (2.0%)	0 (0.0%)	0.498
AC Avastin injection in theatre	0 (0.0%)	2 (2.0%)	0.498
Bleb revision in clinic	3 (3.0%) * **	1 (1.0%)	0.621
Bleb revision in theatre	11 (10.9%) * ***	25 (24.8%) * ** *** ****	0.016
Secondary Cyclodiode treatment	2 (2.0%) *	0 (0.0%)	0.498
Secondary IOP-lowering surgery	2 (2.0%)	0 (0.0%)	0.498
Laser suture lysis	0 (0.0%)	4 (4.0%) *	0.121
Suture removal in theatre	0 (0.0%)	3 (3.0%)	0.246
Total n with complications (excluding hypotony)	32 (31.7%)	46 (45.5%)	0.060
- Including hypotony	59 (48.5%)	66 (65.3%)	0.385
Total n with theatre interventions	17 (16.8%)	31 (30.7%)	0.031

## Data Availability

The datasets used and/or analysed during the current study are available from the corresponding author upon reasonable request.
